# MicroRNA Profiling Response to Acupuncture Therapy in Spontaneously Hypertensive Rats

**DOI:** 10.1155/2015/204367

**Published:** 2015-03-15

**Authors:** Jia-You Wang, Hui Li, Chun-Mei Ma, Jia-Lu Wang, Xin-Sheng Lai, Shu-Feng Zhou

**Affiliations:** ^1^Department of Human Anatomy, College of Fundamental Medical Sciences, Guangzhou University of Chinese Medicine, Guangzhou, Guangdong 510006, China; ^2^Department of Pharmaceutical Sciences, College of Pharmacy, University of South Florida, 12901 Bruce B. Downs Boulevard, MDC 30, Tampa, FL 33612, USA; ^3^Department of Acupuncture and Moxibustion, College of Acupuncture and Moxibustion, Guangzhou University of Chinese Medicine, Guangzhou, Guangdong 510006, China; ^4^Guizhou Provincial Key Laboratory for Regenerative Medicine, Stem Cell and Tissue Engineering Research Center & Sino-US Joint Laboratory for Medical Sciences, Guiyang Medical University, Guiyang, Guizhou 550004, China

## Abstract

MicroRNAs (miRNAs) are a group of endogenous noncoding RNAs that play important roles in many biological processes. This study aimed to check if miRNAs were involved in the response to acupuncture in rats. Microarray analysis was performed to compare the miRNA expression profiles of medulla in spontaneously hypertensive rats (SHRs) treated with or without acupuncture. Our microarray analysis identified 222 differentially expressed miRNAs in the medulla of SHRs treated with acupuncture at taichong acupoint. Among these miRNAs, 23 miRNAs with a significant difference were found in acupuncture-treated SHRs compared to untreated rats. These 23 miRNAs could regulate 2963 target genes which were enriched in at least 14 pathways based on our bioinformatic analysis. miRNA-339, miR-223, and miR-145 were downregulated in the medulla of SHRs compared to normotensive rats. Notably, these miRNAs were upregulated to basal levels in the medulla of SHRs treated with acupuncture at taichong in comparison with SHRs receiving acupuncture at nonacupoint group or SHRs without any treatment. Our findings have revealed significant changes of a panel of selective miRNAs in hypertensive rats treated at taichong acupoint. These data provide insights into how acupuncture elicits beneficial effects on hypertension.

## 1. Introduction

Hypertension, a major risk factor for cardiovascular disease, affects approximately one billion individuals worldwide [1], including 78 million (33%) American adults ≥20 years of age (i.e., it affects one in three adults in the United States) [2]. Although a number of treatment strategies have been developed for this disease, only 75% of hypertensive patients received pharmacologic treatment, and only 53% of those with documented hypertension have their condition controlled to target levels [2]. The major barriers to successful pharmacotherapeutic control of hypertension are poor drug response, adverse side effects, and low patient compliance [3].

Acupuncture originated in ancient China at least 2,500 years ago. Although there is some controversy in mainstream Western medicine, it has become one of the most widely practiced forms of alternative medicine in the world [4]. For example, estimated 3 million American adults receive acupuncture treatment each year [5]. The US Internal Revenue Service approved acupuncture as deductible medical expense in 1973. The National Institute of Health has sponsored a Consensus Development Conference on Acupuncture in 1997 [6]. WHO has approved acupuncture as an alternative therapy for the treatment of at least 64 types of diseases. On November 16, 2010, acupuncture has been listed by the United Nations Educational, Scientific, and Cultural Organization as “Intangible Cultural Heritage.”

Since the 1970s, a number of animal and clinical studies have demonstrated the effectiveness of acupuncture at specific acupoints to lower blood pressure in essential hypertension [7–10]. Unfortunately, the underlying mechanisms through which acupuncture lowers blood pressure remain unclear. Studies have suggested the involvement of nitric oxide (NO) [11], neurotransmitters [12], aldosterone, endothelin, and angiotensin II [13, 14], and acetylcholine, opioids, *γ*-aminobutyric acid, serotonin, and endocannabinoids in the brain all appear to contribute to the acupuncture's antihypertensive effect [15, 16].

The small noncoding microRNAs (miRNAs) have critical functions in the regulation of various critical biological processes such as cell metabolism, proliferation, death, and development [17, 18]. To date, there are more than 2,000 miRNAs reported in humans. A number of studies have found that miRNAs respond to various therapeutic interventions such as pharmacotherapy, physical therapy, and radiotherapy. However, there is no information on the effect of acupuncture therapy on miRNA profiles in animals and humans. The objective of the present study was to determine whether miRNAs were involved in the response to acupuncture therapy in rats. This study is the first to examine the miRNA response in the medulla of rats to acupuncture at the taichong (LR3) point compared to stimulation at a nonacupoint in spontaneously hypertensive rats (SHRs).

## 2. Materials and Methods

### 2.1. Ethics Statement

All animal experiments were performed at the Laboratory Animal Center of Guangzhou University of Chinese Medicine, Guangzhou, China. The procedure was approved by the Ethics Committee of Guangzhou University of Chinese Medicine, Guangzhou, China [permit number: SYXK (Yue) 2008-0085].

### 2.2. Chemicals and Reagents

The 6th generation of miRCURYTM LNA Array (v.16.0) was obtained from KangChen Bio-tech (Shanghai, China), which contains more than 1891 capture probes, covering all human, mouse, and rat microRNAs annotated in miRBase 16.0, as well as all viral microRNAs related to these species. In addition, this array contains capture probes for 66 new miRPlus human microRNAs. All chemicals and reagents used were of analytical grade.

### 2.3. Animals

The experiments were performed with SHRs and Sprague-Dawley (SD) rats provided by Beijing Vital River Laboratory Animals Co., Ltd (Beijing, China). The rats were housed in cages maintained in a temperature- and humidity-controlled room at the Laboratory Animal Center of Guangzhou University of Chinese Medicine, Guangzhou, China. The animals were given a standard diet. The SHRs with confirmed blood pressure ≥ 140 mmHg were included and randomly divided into three groups: (1) acupuncture at taichong group (*n* = 6) treated with acupuncture at LR3 point, (2) acupuncture at nonacupoint group (*n* = 6) treated with acupuncture at nonacupoints, and (3) model group (*n* = 6) untreated with acupuncture throughout the duration of the experiment. In the literature, SD rats have been used as the normotensive controls in comparison to SHRs [19, 20] and thus they were used as normal control group (*n* = 6) in this study.

### 2.4. Acupuncture Procedure

**Figure 1:**
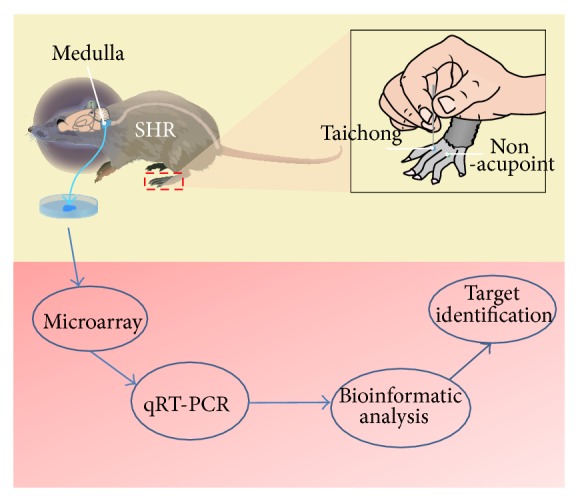
The acupuncture procedure at taichong point and nonacupoint in SHRs. In taichong group of the SHRs, acupuncture was performed at bilateral LR3 located between the 1st and the 2nd metatarsal of dorsal foot, while, in nonacupoint group, acupuncture was done at bilateral nonacupoint located at fossa between the 3rd and 4th metatarsal of dorsal foot.

A number of clinical and animal studies have reported the efficacy of acupuncture at taichong point in reducing hypertension [21–23]. The acupuncture treatment procedure was the same as described previously by us [22]. Briefly, in acupuncture taichong group, acupuncture was performed at bilateral taichong points (LR3) located between the 1st and the 2nd metatarsal of dorsal foot, while, in acupuncture nonacupoint group, acupuncture was done at bilateral nonacupoint located at the fossa between the 3rd and 4th metatarsal of dorsal foot (Figure 1). As previously described [22, 24], the rats were lightly immobilized and the acupuncture needles were inserted to a depth of 3 mm at the appropriate location bilaterally, twisted at a rate of 80 spins per min, and removed afterward. This treatment was given daily (treatment lasting 5 min/day) for 7 consecutive days. After the last acupuncture treatment, rats were anesthetized and their medullas were quickly dissected. The medullas were preserved in liquid nitrogen until analysis.

### 2.5. Microarray Analysis

Total RNA was isolated using TRIzol (Invitrogen, Grand Island, NY, USA) and miRNeasy minikit (QIAGEN) according to manufacturer's instruction. After RNA isolation from the samples, the miRCURY Hy3/Hy5 Power labeling kit (Exiqon, Vedbaek, Denmark) was used according to the manufacturer's guideline for miRNA labelling. One microgram of each sample was 3′-end-labeled with Hy3 fluorescent label, using T4 RNA ligase. After stopping the labeling procedure, the Hy3-labeled samples were hybridized on the miRCURY LNA Array (v.16.0) (Exiqon) according to the manufacturer's manual. The slides were scanned using the Axon GenePix 4000B microarray scanner (Axon Instruments, Foster City, CA, USA). Scanned images were then imported into GenePix Pro 6.0 software (Axon) for grid alignment and data extraction. Replicated miRNAs were averaged and miRNAs in which intensities >50 in all samples were chosen for calculating the normalization factor. The data were normalized using the Median normalization method and after normalization differentially expressed miRNAs were identified through Volcano Plot filtering. In addition, hierarchical clustering was performed using MEV software (v4.6, TIGR).

### 2.6. Bioinformatics Studies

Computational target prediction of miRNAs was conducted by miRDB online searching program (http://mirdb.org/miRDB/). The enriched KEGG pathway for these targets was analyzed in DAVID Bioinformatics Resources 6.7 (http://david.abcc.ncifcrf.gov/).

### 2.7. Quantitative Real-Time PCR (qRT-PCR)

The rat medulla was homogenized in 400 *μ*L of TRIzol reagent (Invitrogen, Grand Island, NY, USA). To avoid contamination with genomic DNA, the RNA samples were treated with RNase-free DNase (Promega, Madison, WI, USA). Reverse transcription was performed using M-MLV reverse transcriptase (Promega, Madison, WI, USA). Real-time RT-PCRs were carried out using an ABI PRISM 7500 Sequence Detection System (Applied Biosystems, Carlsbad, CA, USA) and the SYBR Green PCR Master Mix kit (Toyobo Co., Ltd., Osaka, Japan). The 2^(-ΔΔCt)^ method was used to analyze the RT-PCR data and the ^ΔCt^ of each miRNA was determined relative to U6 RNA and endogenous control miRNA that is robustly and invariantly expressed in all cell types. The experiments were performed in triplicate, and consistent results were obtained.

### 2.8. Statistical Analysis

Data are presented as the mean ± SD. Multiple comparisons were evaluated by one-way analysis of variance (ANOVA) followed by Tukey's multiple comparison procedure, with *P* < 0.05 considered significant. All statistical tests were performed using Prism software version 6.0 (GraphPad Software Inc., Chicago, IL, USA).

## 3. Results

### 3.1. miRNAs Profiling Response to Acupuncture Therapy in SHRs

To reveal miRNAs profiling response to acupuncture therapy in SHRs, microarrays containing 1891 human, rat, and mouse miRNAs were used to examine the expression of miRNAs in medullas after acupuncture treatment at taichong point in SHRs. The analyses were performed using total RNA collected from three rat medullas. Statistical analysis of the miRNA expression data identified 222 miRNAs with significant changes in the expression, with 23 miRNAs having expression levels greater or equal to a 1.5-fold change in SHRs with or without acupuncture treatment (Tables 1 and 2). Among these 23 differentially expressed miRNAs, 8 of 23 miRNAs were significantly upregulated, while 15 of 23 miRNAs were significantly downregulated after acupuncture treatment.

### 3.2. Confirmation of Microarray Profiling Data by qRT-PCR

**Figure 2:**
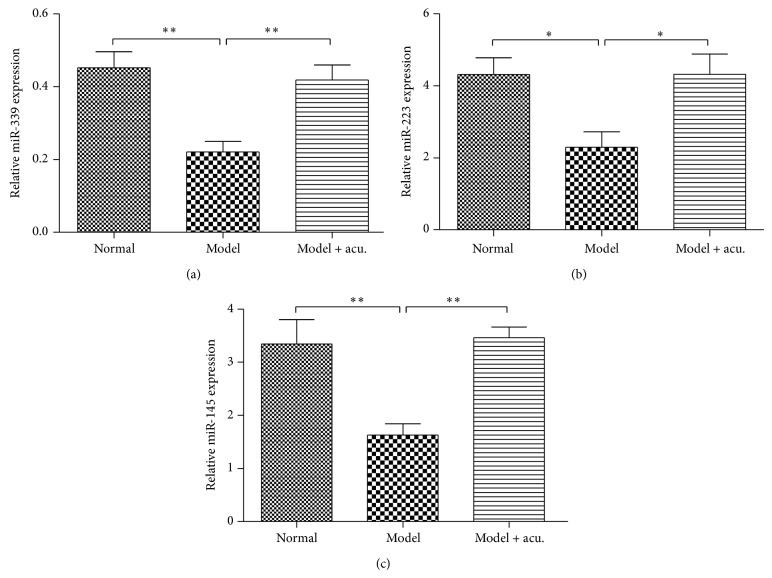
Confirmation of miR-339 (a), miR-223 (b), and miR-145 (c) expression changes in the medulla of SHRs treated with acupuncture at taichong point (model + acu.). The expression level of miR-339, miR-223, and miR-145 in rats was detected using qRT-PCR. The relative expression of these miRNAs was calculated in relation to levels of U6 RNA using the 2^−ΔΔCt^ method. The result was repeated in triplicate independent experiments. ^*^ *P* < 0.05; ^**^ *P* < 0.001.

To validate the microarray profiling data, qRT-PCR was used to confirm the upregulated miRNAs including miRNA-339, miR-223, miR-145, and miR-451. The data showed that miR-339, miR-223, and miR-145 were significantly upregulated in medullas of SHRs treated with acupuncture at taichong point in contrast to the model group untreated with acupuncture (Figure 2). While being compared to the normal control group (healthy SD rats), miR-339, miR-223, and miR-145 were significantly downregulated in model group. There was no significant increase in miR-451 between acupuncture group and model group or normal group (data not shown).

### 3.3. Acupuncture Treatment at Taichong Acupoint Elicits Specific miRNA Expression Changes in SHRs

**Figure 3:**
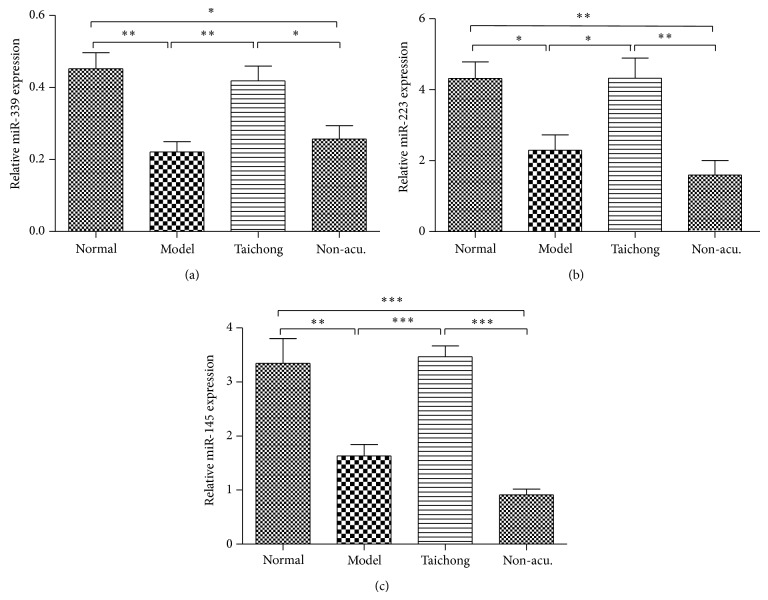
Acupuncture treatment at taichong acupoint elicits specific miRNA expression changes in SHRs. Acupuncture at taichong point (Taichong) significantly increased miR-339 (a), miR-223 (b), and miR-145 (c) expression levels in the medullas of SHRs compared to SHRs with nonacupoint treatment (non-acu.). ^*^ *P* < 0.05; ^**^ *P* < 0.001, ^***^ *P* < 0.001.

Next, we compared the effect of acupuncture at taichong acupoint or at nonacupoint on miRNA expression changes in SHR medulla. The data showed that acupuncture at taichong point significantly increased miRNA-339, miR-223, and miR-145 levels in medullas of SHRs in contrast to the model group, while acupuncture at nonacupoint did not significantly alter miR-339, miR-223, and miR-145 levels compared to the model group. Moreover, compared to acupuncture at nonacupoint, acupuncture at taichong point significantly upregulated the expression of miRNA-339, miR-223, and miR-145 in medullas of SHRs (Figure 3). These data showed significantly different miRNA response to real and sham acupuncture treatments in rats.

### 3.4. Possible Targets and Their Enriched Pathway of Acupuncture-Regulated miRNAs by Bioinformatics Analysis

To explore possible targets and signaling pathways involved in response to acupuncture, we used miRDB to predict the targets of the differentially expressed microRNAs responsive to acupuncture and used DAVID Bioinformatics Resources 6.7 (http://david.abcc.ncifcrf.gov/) to analyze the enriched pathways. From the miRDB online database (http://mirdb.org/miRDB/), we identified 5223 targets for all 23 acupuncture-regulated miRNAs when the target score was set to ≥50. When the target score cut-off was set at ≥60, 2963 possible targets were predicted. We further analyzed the enriched KEGG pathways for these 2963 targets and found 14 pathways, including neurotrophin signaling pathway, pathways in cancer, MAPK signaling pathway, Wnt signaling pathway, Ubiquitin mediated proteolysis, cell cycle, chemokine signaling pathway, oocyte meiosis, tight junction, T cell receptor signaling pathway, axon guidance, and TGF-*β* signaling pathway (Table 3).

## 4. Discussion

In this study, we first found that miRNAs responded to acupuncture treatment in SHRs. Previous studies have demonstrated that physical therapy elicited remarkable miRNA profiling changes in humans [25–28] and mice [29]. Similarly, we have revealed that 222 medullar miRNAs were involved in acupuncture therapy in SHRs. Of these miRNAs, although screened by fold change ≥1.5 and *P* value < 0.05, 23 miRNAs were significantly regulated by acupuncture treatment (Tables 1 and 2). These microarray data were further validated using qRT-PCR. The findings from this study suggest that miRNAs are involved in the therapeutic effects of acupuncture.

Our finding further showed that acupuncture treatment at specific acupoint elicits selective miRNA expression changes in SHRs (Figure 3). Acupoint specificity is the theoretical basis for meridian and acupuncture theory and is also the key factor for the application of effective and individualized acupuncture treatment [30, 31]. However, there is still some controversy about the existence of acupoint specificity [32–37]. Our data showed that acupuncture at taichong acupoint regulated miR-339, miR-223, and miR-145 expression, while acupuncture at nonacupoint failed to affect these miRNAs' expression. Other researches have observed acupoint-specific effect at taichong point in human brain by functional nuclear magnetic resonance imaging technique [38].

Our bioinformatics analysis showed that 23 miRNAs responded to acupuncture and these miRNAs might regulate 2963 targets. These miRNAs have many important physiological and pathological functions. miRNA-339, miR-223, and miR-145 are highly conserved and have multiple targets predicted for them in both humans and rats [39–41]. In addition, many studies have demonstrated that these miRNAs are involved in cell proliferation, apoptosis, and tumor suppressor. For example, miRNA-339 has been shown to have a central role in regulating the expression of intercellular cell adhesion molecule-1 [39]. Similarly, miR-223 has been found to negatively regulate progenitor proliferation and granulocyte differentiation and activation [42]. miR-145 has been proposed to inhibit human embryonic stem cell self-renewal, represses expression of pluripotency genes, and induces lineage-restricted differentiation [41]. miR-145 has also been proposed as a tumor suppressor that can target the 3′-untranslated region of the insulin receptor substrate-1 gene and dramatically inhibit the growth of colon cancer cells [43]. Therefore, acupuncture-responsive miRNAs may contribute to the therapeutic effects of acupuncture in hypertension through various pathways.

**Figure 4:**
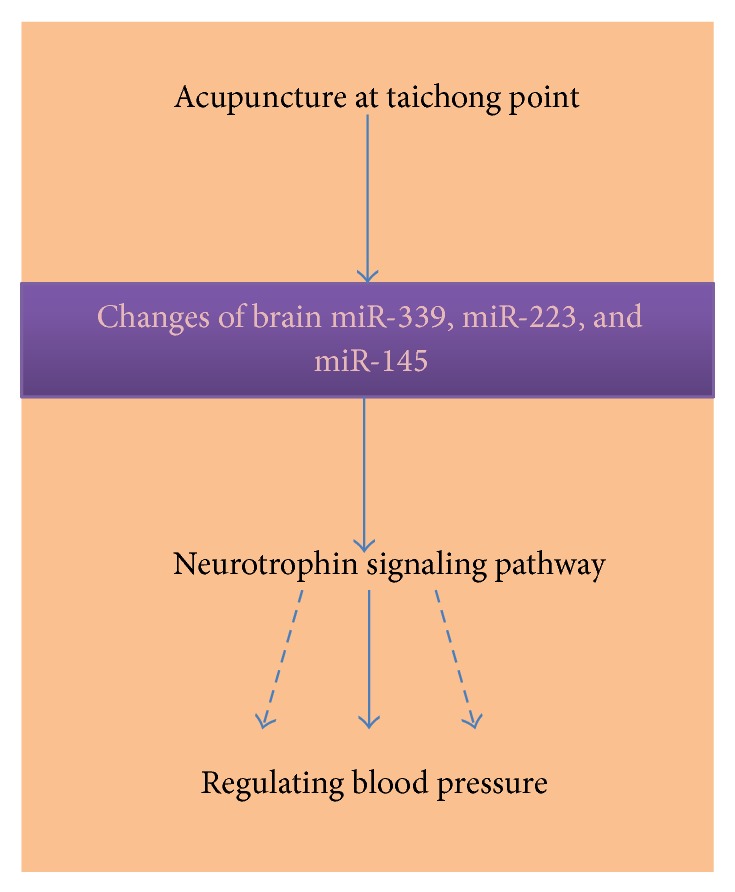
Proposed role of miRNAs in the antihypertensive effects of acupuncture.

Our bioinformatic data also showed that there were 14 pathways regulated by the miRNAs responsive to acupuncture therapy (Table 3). Among these pathways, the neurotrophin signaling pathway plays critical roles in the regulation of brain activities and blood pressure control. Neurotrophins are a family of growth factors that induce the survival, development, and function of neurons [44, 45], which also play an important role in pathogenesis of hypertension [46] and in the neuroprotective activity of acupuncture treatment. The cardiovascular system is tightly controlled by the nervous system; neurons, cardiomyocytes, endothelial cells, and vascular smooth muscle cells are all well regulated by neurotrophins [46–49]. Our data suggest that acupuncture might regulate blood pressure through microRNA-neurotrophin signaling pathway. Studies by us and others have demonstrated that acupuncture activated neurotrophin signaling pathway and elicited neuroprotective activity [50–52]. Recent studies have showed that microRNA can not only regulate neurotrophin expression, but also target their receptors and downstream signaling proteins [53, 54]. For example, miR-206 was found to decrease brain-derived neurotrophic factor level [53, 54] and miR-21 regulated neuron growth factor signaling pathway in rats [55]. miR-592 was shown to modulate the induction of p75 neurotrophin receptor in neuronal ischemic injury [56]. Our data together with data from other groups suggest that acupuncture could modulate various miRNAs which consequently altered the function and activities of critical neuronal and cardiovascular factors contributing to the regulation of blood pressure (Figure 4). The acupuncture-responsive miRNAs are supposed to play important roles in the therapeutic effects of acupuncture treatment for hypertension, but further mechanistic studies are warranted to elicit how these responsive miRNAs contribute to the antihypertensive activity of acupuncture.

In addition, our bioinformatics data predicted that mitogen-activated protein kinase (MAPK) signaling pathway was possibly involved in the antihypertensive activity of acupuncture in rats. The MAPK signaling molecules have been recognized as important mediators in directing cellular responses to a diverse array of stimuli, such as proinflammatory cytokines and exposure to environmental compounds. They regulate gene expression, differentiation, apoptosis and many other cellular processes [57]. Studies have showed that MAPK signaling pathway was activated by acupuncture in the brain [58]. Further functional studies are needed to dissect the role of MAPK signalling pathway in the therapeutic effects of acupuncture.

In summary, our microarray study for the first time identified 222 differentially expressed miRNAs in the medulla of SHRs treated with acupuncture at the taichong acupoint. Among these miRNAs, 23 miRNAs were found to be differentially expressed in acupuncture-treated SHRs compared to untreated control rats. These 23 miRNAs could regulate 2963 target genes based on our bioinformatic analysis. Importantly, our RT-PCR assay has confirmed that miRNA-339, miR-223, and miR-145 were upregulated in SHRs treated with acupuncture at taichong acupoint in comparison with the nonacupoint group. Our findings have demonstrated significant changes of specific and selective miRNAs in rats when taichong acupoint was stimulated. Our data have revealed the specific miRNA profile changes in response to acupuncture treatment and strongly suggest that a selective panel of miRNAs play an important role in the antihypertensive activity of acupuncture therapy.

## Figures and Tables

**Table 1:** miRNAs upregulated by acupuncture in the medulla of SHRs.

Mature miRNAs (rno-miR-)	Fold-change (mean ± SEM)	*P* value	miRBase accession number
339	2.13	0.0214	MIMAT0000583
223	3.34	0.0011	MI0000963
145	3.02	0.0059	MI0000918
451	3.32	0.0097	MI0001731
193	2.88	0.0300	MI0000936
378	2.30	0.0135	MI0003719
423	1.97	0.0277	MI0006145
let-7b^*^	1.70	0.0443	MIMAT0004705

Each experiment was performed in triplicate. The fold change is presented as the mean ± SEM.^*^Indicates antisense mature miRNA.

**Table 2:** miRNAs downregulated by acupuncture in the medulla of SHRs.

Mature miRNAs (rno-miR-)	Fold-change (mean ± SEM)	*P* value	miRBase accession number
7a	0.52	0.0254	MI0000641
9	0.48	0.0491	MI0000838
128	0.45	0.0229	MI0000900
132	0.42	0.031	MI0000905
134	0.59	0.0037	MI0000907
182	0.24	0.0093	MI0006133
335	0.6	0.0475	MI0000612
382	0.44	0.0381	MI0003548
383	0.51	0.0324	MI0003478
434	0.44	0.0155	MI0006147
496	0.59	0.011	MI0012622
135a	0.45	0.0414	MI0000908
136^*^	0.58	0.0131	MIMAT0004733
376b-5p	0.54	0.0052	MIMAT0003195
384-3p	0.57	0.0225	MIMAT0005310

Each experiment was performed in triplicate. The fold change is presented as the mean ± SEM. ^*^Indicates antisense mature miRNA.

**Table 3:** The enriched KEGG pathways of acupuncture-responsive miRNAs in SHRs.

Category	Term	Count	%	*P* value	Benjamini
KEGG_PATHWAY	Neurotrophin signaling pathway	21	0.1	6.60*E* − 02	6.50*E* − 01
KEGG_PATHWAY	Pathways in cancer	51	0.2	6.50*E* − 03	1.60*E* − 01
KEGG_PATHWAY	MAPK signaling pathway	42	0.2	1.90*E* − 02	3.20*E* − 01
KEGG_PATHWAY	Wnt signaling pathway	38	0.2	7.80*E* − 07	1.40*E* − 04
KEGG_PATHWAY	Ubiquitin mediated proteolysis	31	0.1	3.70*E* − 05	3.40*E* − 03
KEGG_PATHWAY	Cell cycle	29	0.1	2.40*E* − 04	8.80*E* − 03
KEGG_PATHWAY	Chemokine signaling pathway	29	0.1	2.40*E* − 02	3.50*E* − 01
KEGG_PATHWAY	Calcium signaling pathway	28	0.1	7.50*E* − 02	6.40*E* − 01
KEGG_PATHWAY	Oocyte meiosis	27	0.1	1.60*E* − 04	7.40*E* − 03
KEGG_PATHWAY	Tight junction	22	0.1	5.20*E* − 02	5.90*E* − 01
KEGG_PATHWAY	T cell receptor signaling pathway	21	0.1	1.70*E* − 02	3.20*E* − 01
KEGG_PATHWAY	Endocytosis	42	0.2	7.70*E* − 05	4.70*E* − 03
KEGG_PATHWAY	Axon guidance	21	0.1	7.00*E* − 02	6.40*E* − 01
KEGG_PATHWAY	TGF-beta signaling pathway	20	0.1	2.50*E* − 03	7.40*E* − 02

Thresholds: count cut-off ≥20; EASE <0.1.
